# The two tryptophans of β2-microglobulin have distinct roles in function and folding and might represent two independent responses to evolutionary pressure

**DOI:** 10.1186/1471-2148-11-159

**Published:** 2011-06-10

**Authors:** Sara Raimondi, Nicola Barbarini, Palma Mangione, Gennaro Esposito, Stefano Ricagno, Martino Bolognesi, Irene Zorzoli, Loredana Marchese, Cristina Soria, Riccardo Bellazzi, Maria Monti, Monica Stoppini, Mario Stefanelli, Paolo Magni, Vittorio Bellotti

**Affiliations:** 1 Department of Biochemistry, University of Pavia, via Taramelli 3b, 27100 Pavia, Italy; 2 Department of Computer Engineering and Systems Science, University of Pavia, via Ferrata 1, 27100 Pavia, Italy; 3 Department of Biomedical Sciences and Technologies, University of Udine, P.le Kolbe 4, 33100 Udine, Italy; 4 Department of Biomolecular Sciences and Biotechnology, CNR-INFM and CIMAINA, University of Milano, Via Celoria 26, 20133-Milan, Italy; 5 Department of Internal Medicine and Medical Therapy, University of Pavia, viale Golgi 19, 27100 Pavia, Italy; 6 Department of Organic Chemistry and Biochemistry, University of Naples "Federico II" and CEINGE-Biotecnologie Avanzate, via Cinthia 6, 80126 Naples, Italy

## Abstract

**Background:**

We have recently discovered that the two tryptophans of human β2-microglobulin have distinctive roles within the structure and function of the protein. Deeply buried in the core, Trp95 is essential for folding stability, whereas Trp60, which is solvent-exposed, plays a crucial role in promoting the binding of β2-microglobulin to the heavy chain of the class I major histocompatibility complex (MHCI). We have previously shown that the thermodynamic disadvantage of having Trp60 exposed on the surface is counter-balanced by the perfect fit between it and a cavity within the MHCI heavy chain that contributes significantly to the functional stabilization of the MHCI. Therefore, based on the peculiar differences of the two tryptophans, we have analysed the evolution of β2-microglobulin with respect to these residues.

**Results:**

Having defined the β2-microglobulin protein family, we performed multiple sequence alignments and analysed the residue conservation in homologous proteins to generate a phylogenetic tree. Our results indicate that Trp60 is highly conserved, whereas some species have a Leu in position 95; the replacement of Trp95 with Leu destabilizes β2-microglobulin by 1 kcal/mol and accelerates the kinetics of unfolding. Both thermodynamic and kinetic data fit with the crystallographic structure of the Trp95Leu variant, which shows how the hydrophobic cavity of the wild-type protein is completely occupied by Trp95, but is only half filled by Leu95.

**Conclusions:**

We have established that the *functional *Trp60 has been present within the sequence of β2-microglobulin since the evolutionary appearance of proteins responsible for acquired immunity, whereas the *structural *Trp95 was selected and stabilized, most likely, for its capacity to fully occupy an internal cavity of the protein thereby creating a better stabilization of its folded state.

## Background

Over the past few years, the investigation of β2-microglobulin (β2-m) amyloidogenesis has shed light on the pathogenesis of Dialysis Related Amyloidosis (DRA) [1] and has provided general information on the mechanism of structural transition of globular proteins into amyloid fibrils [2-4]. Single amino acid substitutions in the protein sequence enabled us to demonstrate the pivotal role of the two tryptophan (Trp) residues in the function and amyloidogenic propensity of this protein. Moreover, we have recently discovered a functional role of Trp60 in promoting the intermolecular association of β2-m with the MHCI heavy chain and in enhancing the conformational flexibility of the loop between strands D-E and the N-terminal stretch [5]. This conformational flexibility involving Trp60 is necessary for the optimal binding of β2-m to the MHCI heavy chain, although, at the same time, this increases the intrinsic tendency of the protein to self aggregate.

In contrast, Trp95 is buried in the hydrophobic core of the protein and is apparently essential for its stability; however Trp95 does not contribute directly to the binding of the MHCI heavy chain. Tryptophan is a relatively rare amino acid within a protein sequence and its large hydrophobic surface area containing the heterocyclic ring system has a unique role in protein folding and function [6]. These two properties drive protein remodelling during evolution wherein a trade-off exists between mutations that endow better protein function regardless of protein fitness and compensatory mutations that improve stability [7]. The two Trp residues of β2-m represent two examples of how this amino acid can affect protein structure or function; therefore, we analysed the evolutionary tree and the conservation of the two residues in vertebrates expressing MHCI molecules. We have discovered that Trp60 is highly conserved, whereas, in some of the most basal taxa, Leu is present in position 95. To understand the possible positive effects of the replacement of Leu95 with Trp we investigated the structural impact of this mutation on human β2-m.

## Results and Discussion

### Conservation and phylogenetic analysis

**Figure 1:**
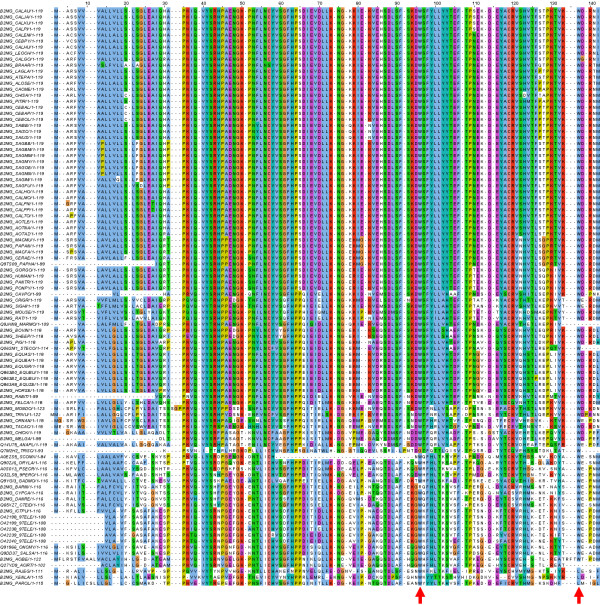
**Multiple alignments obtained using the CLUSTALW algorithm**. The multiple alignments are displayed with the JalView applet, using the CLUSTALX colour mode. Two arrows indicate the amino acids of interest.

**Figure 2:**
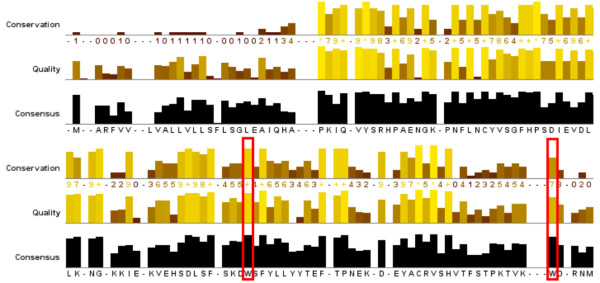
**Conservation analysis performed on the multiple alignment with the JalView applet**.

We used the FamFetch tool with the entry name B2MG_HUMAN to search the HOVERGEN database. The retrieved family, HBG006197, consisted of 130 protein sequences from 96 different species with *Gnathostomata *as a common ancestor. We selected a single sequence for each species and the CLUSTALW algorithm was used to align the resulting 96 proteins (Figure 1). A simple conservation analysis of the amino acids based on this multiple alignment allowed us to compute conservation, quality and consensus annotation for a specific region (Figure 2). In the multiple alignment, Trp60 and Trp95 of mature β2-m are located at positions 96 and 137, respectively, and both show a consensus over 90% (97% and 94%, respectively). This observation represents the first evidence of the relevance of the two amino acids. However, the conservation and the quality annotation show that the chemistry and the quality of the conservation is high for Trp60 (10 and 203.034, respectively), but quite low for Trp95 (7 and 173.665) suggesting a high probability of mutation for Trp95. As described previously, while Trp60 is solvent-exposed and essential for promoting intermolecular association, Trp95 is buried in the hydrophobic cavity of the protein delimited by residues Ser11, Asn21, Leu23, Phe70, Pro72 and Tyr78. This analysis shows that all these amino acids have a high percentage of consensus, as well as high values of conservation and quality annotation: Ser11 (position 42): 91%, 9, 208.772; Asn21 (position 53): 98%, 10, 209.680; Leu23 (position 55): 95%, 10, 214.541; Phe70 (position 106): 95%, 10, 205.723; Pro72 (position 109): 98%, 10, 215.842; Tyr78 (position 117): 89%, 9, 194.226.

**Figure 3:**
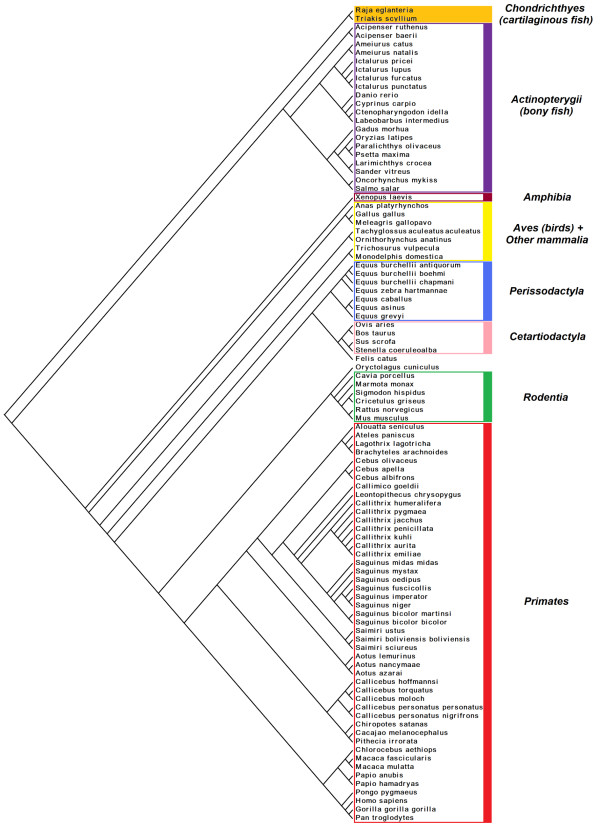
**Species tree extracted using the NCBI Taxonomy Browser**. The main taxonomic groups are highlighted.

The phylogenetic tree was constructed using four different methods: Neighbor-Joining (NJ), Maximal Parsimony (MP), Minimum Evolution (ME) and Bayesian Inference (BI). The taxonomy of the organisms was considered as a species tree (Figure 3) with *Triakis scyllium *and *Raja eglanteria *as the most basal, both belonging to *Chondrichthyes *(cartilaginous fishes), which was also the most divergent class. The distinction between those two species can only be based on the fossil records of the corresponding genus; in fact the *Triakis *genus is dated from the Palaeocene 65.5-55.8 Ma (million years ago), whereas the *Raja *genus is from the Maastrichtian (70.6-65.5 Ma). For this reason, we selected *Raja eglanteria *as a unique and the most basal taxon. Therefore, we re-rooted the gene trees by selecting as root the *Raja eglanteria *protein, B2MG_RAJEG. Moreover, the sequence from *Triakis scyllium *has limited similarity with all the other family members, so the alignment and the subsequent conclusions on the conservation would have limited reliability. It is worth noting that the most basal species, in which β2-m is present, belong to cartilaginous fishes; therefore, we can approximately date the appearance of the protein at about 500 Ma (455 Ma for the fossil record and 528 for molecular time, estimation based on a large scale multiple genes alignment) [8]. Therefore, we can assume that the appearance of β2-m in vertebrates is coincident with the appearance of adaptive immunity and the expression of MHCI and related molecules.

**Figure 4:**
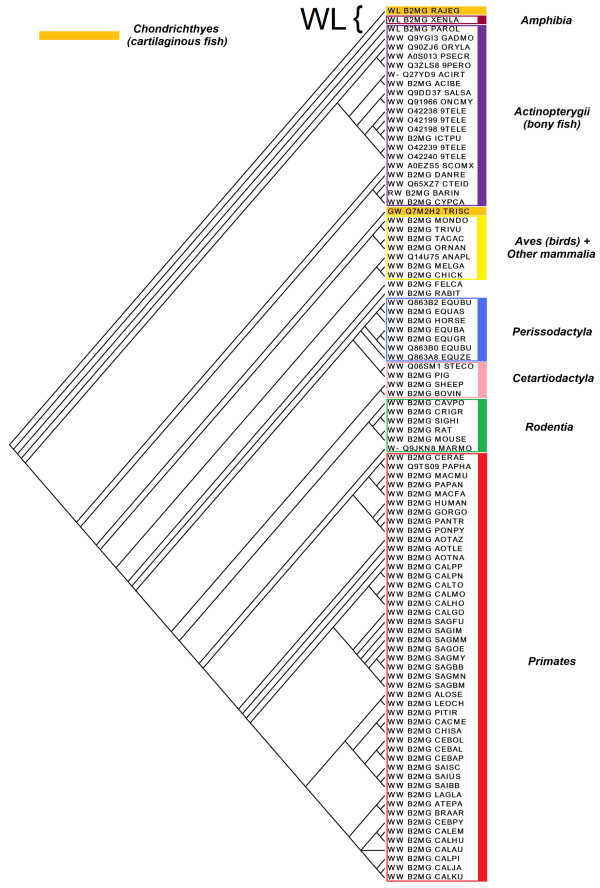
**Gene tree built using the Bayesian Inference method with MrBayes 3.1.2**. The main taxonomic groups are highlighted. Amino acids at positions 96 and 137 in the multiple alignments are indicated; the group characterized by Trp and Leu is indicated.

The evolution of β2-m is similarly represented by the four re-rooted gene trees and the species tree. The topology of these four gene trees was compared with the species tree by computing the Robinson-Foulds metric. The gene tree, which better follows species evolution, was constructed by BI (difference equals to 116) (Figure 4) and was considered as the reference gene tree. A simple reconciliation analysis performed on 20 representative species confirmed the topology agreement between gene and species trees (Additional file 1). The main differences between gene and species trees are the positions of the sequences from the unique *Amphibia *organism, *Xenopus laevis *and *Paralichthys olivaceous *(a bony fish). Choi *et al. *demonstrated that the β2-m of *Paralichthys olivaceous *(flounder) is very similar to that of other sea-fish, such as *Raja Eglanteria*, but it is phylogenetically distant from other β2-m proteins belonging to fishes (e.g. *Danio rerio *(Zebrafish), *Ictalurus punctatus *(Catfish), *Oncorhynchus mykiss *(Trout) and *Ctenopharyngodon idella *(Carp) [9]. The reconciled trees show few duplications of the gene that explains the difference between species and gene trees (Additional file 2); it is interesting that the first duplication seems to be influenced by the presence of Leu or Trp95. Similar conclusions can be achieved by analysing the more complex tree obtained from the reconciliation of the whole gene and species trees (Additional file 3).

In the gene tree reported in Figure 4, the name of each related protein is preceded by the one letter code of the two residues associated with the 96 and 137 positions corresponding to Trp60 and Trp95, respectively, in mature β2-m. The comparison between the species and the gene trees allows us to analyse the phylogenetic evolution of positions 60 and 95. Most of the sequences contain tryptophan. Only a few sequences have different amino acids in these positions. In particular, a pseudo-cluster of three proteins, derived from *Chondrichthyes *(cartilaginous fishes), *Actinopterygii *(bony fishes) and *Amphibia *(amphibians), contains Trp at position 60 and Leu at position 95 instead of Trp. It is worth noting that the gene tree clearly shows an evolutionary divergence of β2-m in warm-blooded vertebrates and fish [8]. Thus, the phylogenetic analysis shows that, in some species belonging to lineages like cartilaginous fishes and amphibians, there is a different hydrophobic residue, leucine, at position 95. Most likely, Trp95 is the result of a diversification process arising during the evolution of the adaptive immune response system [8]. The evidence that all species encoding MHCI contain Trp60 demonstrates the essential role of this residue. These results were confirmed by performing both joint and marginal reconstruction of the ancestral sequence: this sequence has a Trp at position 60 and a Leu at position 95 with good joint log likelihood values at these positions; moreover, good confidence of the reconstruction at these sites was estimated by marginal probabilities (94% for Trp and 69% for Leu).

### Experimental analysis of the role of the two Trp residues in the structure and function of β2-m

**Figure 5:**
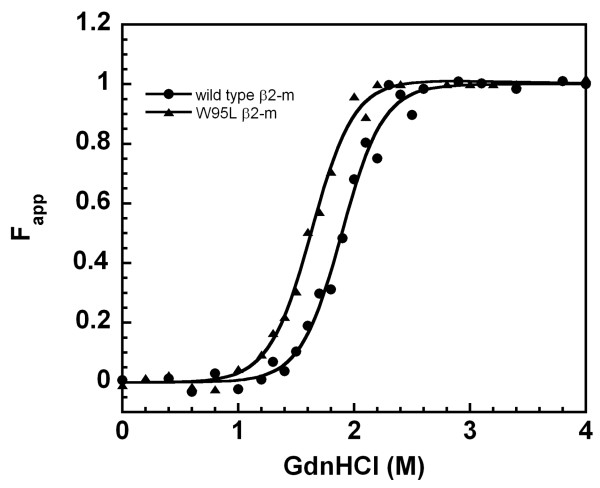
**Equilibrium GdnHCl denaturation curves of wild-type and Trp95Leu β2-m**. GdnHCl denaturation of protein was monitored at pH 7.4 and 20°C by far UV circular dichroism (215 nm). Ellipticities were converted to the apparent unfolded fraction as described in the Methods section.

**Figure 6:**
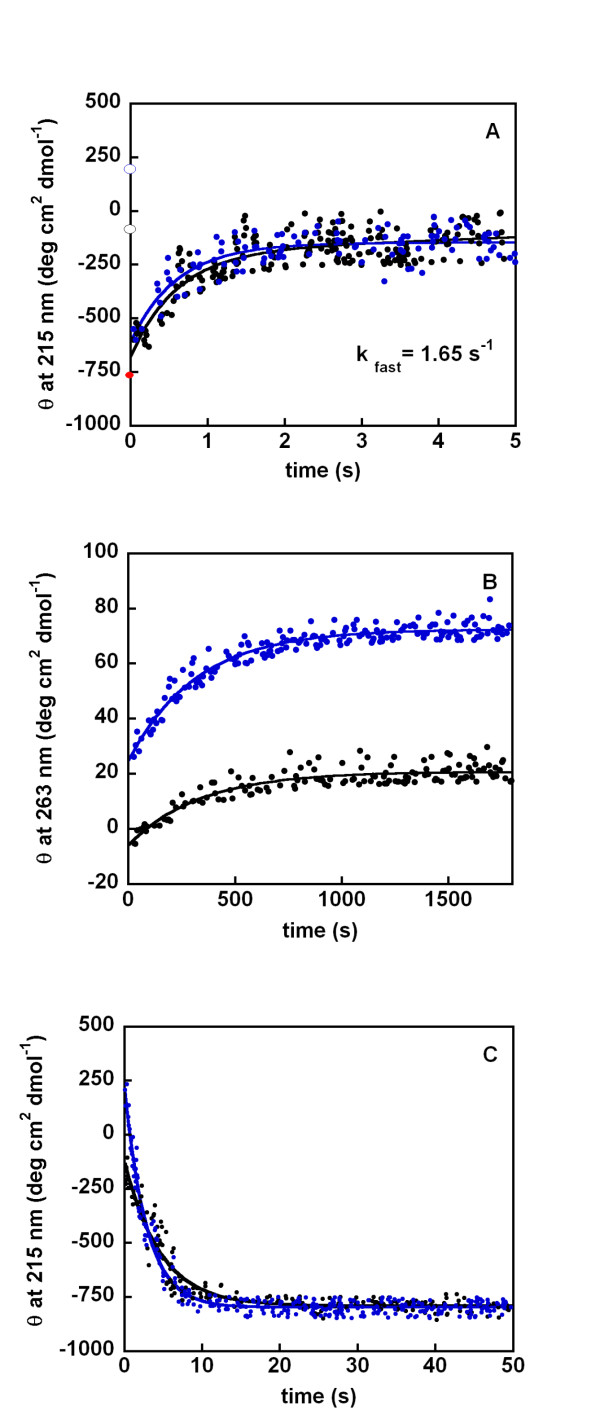
**Change of mean residue ellipticity during the fast (A) and slow (B) phase of folding and unfolding (C) of wild-type (black) and Trp95Leu β2-m (blue)**. The kinetic traces were acquired at pH 7.4 and 30°C in the presence of 0.3 M and 5.1 M GdnHCl for folding and unfolding, respectively. Ellipticity at 215 nm was monitored for the fast phase of folding and unfolding, whereas change in 263 nm mean residue ellipticity was used in the slow phase of folding (B). Mean residue ellipticities at 215 nm of denatured protein (filled red circle), wild-type (empty black circle) and Trp95Leu β2-m (empty blue circle) are reported in (A) for comparison.

We have previously shown that the invariance of Trp in the evolutionary tree can be rationalized by the essential role of Trp60 in the binding of the MHCI complex [5]. In fact, the indole ring of Trp60 fits perfectly into a low-polarity niche within the heavy-chain association interface (PDB entry 2BSS); it provides an interchain hydrogen bond that is highly conserved in complexes with MHCI and CD1 [10]. Trp60 is completely exposed to solvent in its role of anchoring β2-m to MHCI, while Trp95 is fully buried in the hydrophobic core of the protein and its replacement with a small non-hydrophobic amino acid, such as glycine, affects the overall structure and stability of β2-m [5]. The discovery, through phylogenetic analysis, that three basal species from the classes *Chondrichthyes*, *Actinopterygii *and *Amphibia *display a Leu in position 95 prompted us to produce a Leu95 variant of human β2-m to analyse the effect of this replacement on the stability and dynamics of β2-m folding. Since the main intrinsic fluorescence of β2-m originates from Trp95, we have investigated its folding stability and kinetics using circular dichroism (CD). Figure 5 reports the comparative analysis of guanidinium chloride (GdnHCl) unfolding of the Trp95Leu variant and wild-type β2-m, monitored through analysis of CD spectra and measurement of ellipticity at 215 nm. The presence of Leu at site 95 induces a destabilization of 1.0 kcal/mol, with a C_m _shift from 1.9 to 1.65 M GdnHCl. Stopped flow CD apparatus was required to monitor the kinetics of folding and unfolding (Figure 6) on the millisecond to second time-scale, whereas a conventional CD spectropolarimeter was used to determine slow changes in the minute time-scale. The kinetics of folding of β2-m can be dissected into three phases: a very fast phase occurring in the lag time of the measurement (< 5 ms), followed by a fast (Figure 6, panel A) and a slow phase (Figure 6, panel B). The presence of Leu does not affect the measurable phase of refolding; the hydrophobicity of the Leu side chain guarantees the collapse of the hydrophobic core of the molecule and further organization of the secondary structure with the same efficiency as Trp. In fact, the rate constant of the fast phase of folding, monitored at 215 nm, was 1.65 (± 0.8) s^-1 ^for both proteins. The full recovery of native structure was measured in the near UV region and kinetic traces were acquired at the representative wavelength of 263 nm. In each case, an exponential phase was detected and the same resulting rate constant, 0.003 (± 0.0004) s^-1^, was determined for both proteins. The lack of the Trp95 indole ring affects mainly the folding stability; in fact the unfolding kinetics is affected by the Trp-Leu replacement. Figure 6 (panel C) shows that upon exposure to 5.1 M GdnHCl, Leu95 β2-m unfolds at a higher rate constant, 0.4 (± 0.06) s^-1 ^compared with the wild-type protein, 0.22 (± 0.03) s^-1^, in perfect agreement with the reduced stability of the variant at equilibrium.

**Figure 7:**
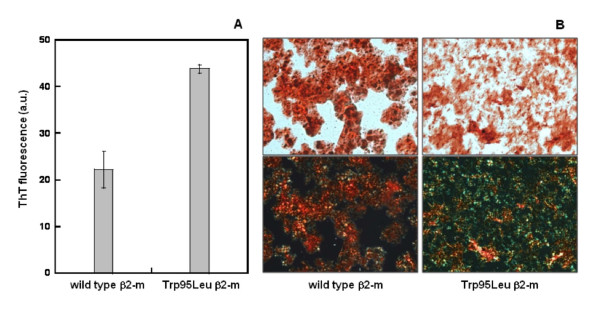
**Comparative analysis of amyloid fibril formation by wild-type and Trp95Leu variant**. A) Thioflavin assay of fibrils formed after incubation for 72 hours. B) Upper panels: light microscopy image at 10 × magnification of Congo red stained wild-type and Trp95Leu variant β2-m after incubation for 72 hours (same material as in Figure 7A). Lower panels: polarized light microscopy image of Congo red stained β2-m amyloid fibrils. The apple green birefringence is characteristic of amyloid.

A reduced folding stability of β2-m variants generally correlates with a higher propensity for self aggregation and formation of amyloid fibrils [5]. In Figure 7 we report the results of the fibrillogenesis test carried out at neutral pH and 20% trifluoroethanol (TFE), where we compared the amount of amyloid fibrils produced from wild-type and the Leu95 variant of β2-m. Both the thioflavin assay and the classical green birefringence typical of amyloid clearly confirm the hypothesis of a higher amyloidogenic propensity of the variant carrying this ancestral amino acid substitution.

**Figure 8:**
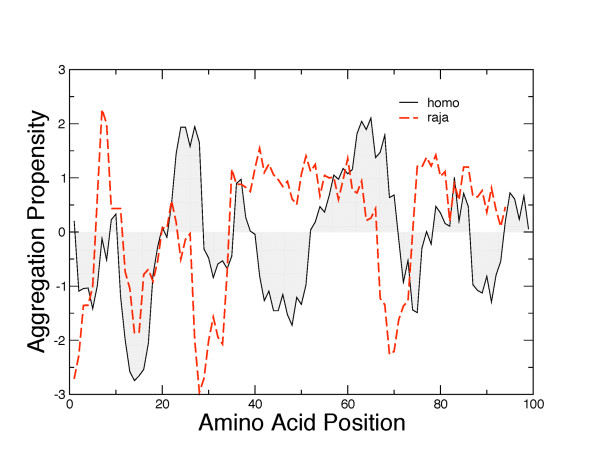
**Zagg aggregation propensities of human (black line) and raja (red line) β2-m calculated with the Zyggregator method **[11]. Zagg represents the intrinsic propensity to form amyloid aggregates calculated from the unfolded state.

The experimental data are in good agreement with the prediction of the aggregation propensity, calculated according to Tartaglia et al [11]. In particular, the carboxy terminal end of the protein around position 95 shows a significantly lower aggregation propensity for human β2-m compared with that of *Raja *β2-m. (Figure 8).

### Crystal structure of the Trp95Leu β2-m mutant

**Figure 9:**
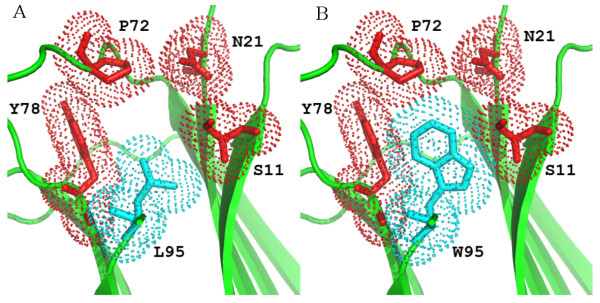
**Comparison between β2-m cavity accommodation of residue 95 in Trp95Leu mutant (A) and wild-type (PDB code **1LDS**) (B)**. Residues Ser11, Asn21, Pro72 and Tyr78 are shown in red as sticks and space-fill dots. Residue 95 is shown in cyan.

The Trp95Leu mutant was crystallised according to the protocol used for the Trp60Val β2-m mutant [12], yielding crystals belonging to the same space group and with the same crystal packing observed for the β2-m DE loop mutants, as recently reported [5,12,13]. Trp95Leu mutant crystals diffracted to a resolution of 1.57 Å. In accordance with the single residue mutation, the Trp95Leu mutant crystal structure is very similar to that of wild-type β2-m (R.S.M.D. 0.55 Å, calculated over 97 Cα pairs), and the mutated Leu95 side chain matches the location of the Trp95 indole ring in the wild-type protein (Figure 9 and Table 1). On the other hand, the cavity created by residues Ser11, Asn21, Leu23, Phe70, Pro72 and Tyr78, which perfectly fits the bulky side chain of Trp95 in wild-type β2-m, is only half filled by Leu95 (Figure 7). Given the hydrophobicity of the cavity, however, no water molecules appear to fill the gaps, leaving the cavity partially empty. Such a condition is unfavourable for protein stability and, together with the loss of an H bond, certainly contributes to the lower stability observed for the Trp95Leu mutant. In fact, Trp95 can establish an H bond with the carbonyl of Asp96 (wild-type β2-m code 2YXF) or with the carboxyl group of Met99 (Trp60Gly mutant code 2Z9T). Consequently, the presence of the mutated Leu95 residue is reflected by increased flexibility of the downstream residues. The whole 96-99 segment displays poor electron density, and could only be modelled with 0.5 occupancy. Conversely, all previously reported mutants that were crystallized under the same conditions and whose crystals were isomorphous with those of the Trp95Leu mutant, consistently showed very good electron density for the C-terminal segment [5,12,13].

**Table 1:** Data collection and refinement statistics for the β2-m Trp95Leu variant

	β2-m Trp95Leu
Beam line	ESRF ID14-1

Space group Unit cell edges (Å, °)	a = 77.41, b = 28.98, c = 54.45, β = 121.7

Resolution (Å)	26.6-1.57

R merge* (%)	5.6 (25.1)

I/σI	17.0 (5.4)

Completeness (%)	99.8 (99.4)

Redundancy	4.4 (4.3)

Unique reflections	14630 (2118)

**REFINEMENT**	

R work** (%)	15.2

R free (%)	20.5

Number of atoms	998

Protein	908

Water	72

Ramachandran plot	

Most favoured region	97.8%

Allowed region	2.2%

Values in parenthesis are for the highest resolution shell.

* R merge = Σ | I - < I > |/Σ | I | where I is the observed intensity and < I > is the average intensity.

** R work = Σ_hkl_||Fo|-|Fc||/Σ_hkl_|Fo|for all data except 5% which were used for R free calculation.

Another unexpected structural difference observed in the Trp95Leu mutant is located in the DE loop. Such a loop was observed in several conformations in different β2-m structures and/or variants [12], but it always displayed high quality electron density. In the Trp95Leu isoform the electron density for the DE loop is of poor quality; such a loop was modelled in two alternative conformations, with residual electron density suggesting an even higher number of conformations. Relative to the DE loop, residue 95 is located on the opposite pole of the β2-m tertiary structure; in the crystal packing, however, the two regions, from spatially neighbouring molecules, fall close to each other, hence the observed flexibility of the C-terminal region may affect the conformational flexibility of the DE loop.

## Conclusions

Our study indicates that Trp60 of β2-m is highly conserved, which is consistent with its essential role in the binding of β2-m to the heavy chain of MHCI. In contrast, Trp95 is buried in a hydrophobic cavity and contributes to protein stability and in some species is replaced by Leu (for example, within cartilaginous fish and amphibians). Our data suggest that the divergence between Trp or Leu at position 95 and the subsequent selection of Trp in the very large majority of species is based on a significant thermodynamic stabilization of the protein which also limits the intrinsic propensity of β2-m to make amyloid fibrils [3]. Such an effect can be explained by the perfect fit of Trp95 in the hydrophobic cavity delimited by the side chains of Ser11, Leu23, Phe70, Pro72, Tyr78 and Arg97 (Figure 9) in the core of β2-m.

Moreover, because the tendency to self-aggregate correlates with intracellular sequestration and degradation through quality control, it is plausible that a better stability and a lower aggregation propensity have favoured a much better yield of correctly folded β2-m.

## Methods

### Definition of the protein family

A protein (or gene) family consists of all the sequences homologous to the protein of interest. To retrieve these sequences, one approach is a simple similarity search against current databases, performed using the Blast algorithm. A more accurate approach is based on the databases of homologous genes, which are available as HOVERGEN, HOGENOM and HOMOLENS [14-17].

A BLASTP search of our protein of interest (human β2-m) against the NCBInr database allowed us to retrieve homologous sequences only from vertebrates. As a consequence we decided to consider HOVERGEN (Homologous vertebrate gene families) as a reference database [18-20]. HOVERGEN contains all vertebrate protein sequences from the UniProt Knowledgebase (Swiss-Prot and TrEMBL) grouped according to similarity scores; the results of this clustering have been processed to avoid inconsistencies. We identified the human β2-m family using the FamFetch tool by searching HOVERGEN release 48 (May 2007) with the entry name of the protein of interest, i.e. Uniprot entry name B2MG_HUMAN.

Following the approach used by Tourasse [14], the orthology of sequences was assessed by examination of the phylogenetic tree of the family provided in HOVERGEN. When multiple sequences were present for a given species, only the sequence more similar to the remaining sequences was kept, while avoiding the selection of sequences annotated as a fragment; in this way the possibility of multiple substitutions at the same site is minimized.

### Multiple alignment and conservation analysis

We used HOVERGEN to provide a multiple alignment for every family obtained by MUSCLE [21]; however, we also performed a further analysis using CLUSTALW [22,23], which is the most commonly used algorithm for this task [16,17,24]. Sequences were aligned with CLUSTALW version 2.0.12 using the default parameters (except for gap open penalty = 1).

A simple conservation analysis was performed using the tool, JALVIEW [25,26]. Three measures were computed: (i) alignment conservation annotation measures the number of conserved physicochemical properties for each column of the alignment applying the approach used in the AMAS method [27]; (ii) alignment quality annotation is a measure of the likelihood of observing mutations (if any) in a particular column of the alignment. The quality score is calculated for each column in the multiple alignment by summing, for all mutations, the ratio of the two BLOSUM62 scores for a mutation pair and each residue's conserved BLOSUM62 score (which is higher); (iii) alignment consensus annotation is composed by the most frequent residue per column of the alignment, hence it represents the percentage frequency of these amino acids.

### Phylogenetic tree

Phylogenetic (or gene) tree analysis was based on multiple alignment using four different methods for tree reconstruction: Neighbor-Joining, Maximal Parsimony, Minimum Evolution and Bayesian Inference [28,29] The first three methods were applied using MEGA 4 [30-32]. We used the Jones-Taylor-Thornton (JTT) amino acid substitution matrix, which is based on the same counting approach as the PAM matrix but uses a much enlarged database [33]; it is widely used for phylogenetic analysis [16,17] and is the most suitable choice for vertebrates [34]. Statistical reliability of the nodes was assessed by bootstrap analysis (1000 replications) [35]. BI was performed using MrBayes 3.1.2 [36,37]. Four Markov chains of 30,000 generations were run (after a 5000-generation burn-in) at the default temperature (0.2) with a random starting tree and a sampling frequency of 10. A mixed substitution model was set by allowing model jumping between nine fixed-rate amino acid models.

The four phylogenetic trees (or gene trees) were re-rooted by selecting as root the gene belonging to the most basal species. The species tree of the organisms of interest was retrieved to select the best root. This species tree was also useful to analyse the evolution of the protein of interest (and specific amino acids) in comparison with species evolution. The taxonomy of the organisms is usually considered as a species tree. In particular, we used NCBI Taxonomy Browser to extract the taxonomy tree of the analysed organisms [38,39]. It employs a database of all the organisms represented in the NCBI sequence database, and can automatically build a species tree using organisms selected by the user. When the root was selected, all the trees were re-rooted exploiting the functionalities of MEGA software. To evaluate how the protein follows species evolution, a comparison of the topology between the gene tree and the species tree was carried out using the Robinson-Foulds metric implemented in MrBayes [40]. To evaluate the topology agreement and to highlight the importance of the amino acids under investigation, a simple reconciliation analysis was performed by GeneTree 1.3.0 [41]. For simplifying the interpretation of the results, we considered only 20 proteins corresponding to the most representative species. Reconciliation analysis was also performed on the whole gene and species tree using the Notung 2.6 program [42].

Two specific amino acids of the reference protein sequence were the subjects of the evolutionary analysis (i.e. Trp60 and Trp95); therefore, we stored the positions of these amino acids. We extracted the amino acids localized in the stored positions within each sequence by considering the multiple alignments obtained as reference. In particular, we added the information related to these amino acids by concatenating the one letter code in the entry name of the proteins to generate a close-up of the amino acid evolution. To perform this procedure we used Phytreetool, contained in the Bioinformatics Toolbox 2.5 of MATLAB Version 7.4.0.287 (R2007a). Moreover, a formal ancestral sequence reconstruction was performed using the FASTML tool, assuming a gamma distribution of rates among sites [43].

### Equilibrium denaturation experiments

Thermodynamic stability was determined by monitoring the change in far-UV circular dichroism signals at 215 nm of protein samples equilibrated at increasing concentrations of GdnHCl at 20°C. Measurements were performed with a Jasco 710 spectropolarimeter equipped with a temperature control system, using a 1 mm path-length cell. The protein concentration was 200 μg/ml in 10 mM sodium phosphate buffer, pH 7.4 with GdnHCl concentrations ranging from 0 to 4 M. The change in ellipticities was analysed as a function of denaturant concentration according to the method described by Santoro and Bolen, to yield the free energy of unfolding in the absence of denaturant and the GdnHCl concentration at half denaturation [44]. Experimental data were converted to the unfolded fraction using f_U _= (y - y_N_)/(y_U _- y_N_), where y is the ellipticity value at a given denaturant concentration, and y_N _and y_U _are the values of the native and unfolded protein, respectively, extrapolated from the pre- and post- transition base lines defined by the Santoro and Bolen equation [44].

### Folding kinetics followed by far and near-UV Circular Dichroism

A Bio-Logic SFM 3 stopped-flow device coupled to a Jasco 710 spectropolarimeter was used to monitor the rapid changes of ellipticity occurring during folding and unfolding of proteins. Stopped flow traces were monitored at 215 nm with a 2 mm path-length FC-20 cell. All the experiments were performed at 30°C in 10 mM sodium phosphate buffer pH 7.4 with 0.3 mg/ml final protein concentration. The unfolding reactions were performed using a 10-fold dilution of a denaturant free solution of protein at 3 mg/ml with 8 volumes of buffer containing 6 M GdnHCl and one volume of 3 M GdnHCl to yield a final denaturant concentration of 5.1 M. The refolding experiments were carried out using a tenfold dilution of protein samples at 3mg/ml unfolded in 3 M GdnHCl into 10 mM sodium phosphate buffer, pH 7.4.

Slow changes during folding were monitored with a Jasco 710 spectropolarimeter in the near UV region using a 10 mm path-length cell at 30°C and a wavelength of 263 nm. For each protein, one volume of 3 mg/ml denatured at equilibrium in 3 M GdnHCl, was mixed with nine volumes of 10 mM sodium phosphate buffer, pH 7.4. In each case, wavelengths were chosen in regions of maximum spectral change between the native and the denatured protein forms previously recorded in the steady-state CD spectra (data not shown). All the kinetic traces acquired as a function of time from unfolding and folding experiments were fitted as previously described [45].

### Amyloid fibril formation

Wild-type and Trp95Leu β2-m amyloid aggregation was carried out by incubating 100 μM of protein at 37°C in 50 mM phosphate buffer and 100 mM NaCl, pH 7.4, in the presence of 20% (v/v) TFE [46]. β2-m fibril seeds (20 μg/ml) were added to the samples to prime fibrillogenesis.

Amyloid formation was evaluated by both microscopic analysis of the samples stained with Congo red as described previously [47] and by the thioflavin T (ThT) assay according to LeVine [48]. ThT (Sigma-Aldrich St. Louis, MO 63103, USA) concentration was 10 μM in 50 mM glycine-NaOH buffer, pH 8.5. Measurements were recorded from three independent experiments in triplicate using a Perkin Elmer LS50 spectrofluorometer with excitation and emission wavelengths at 445 and 485 nm, respectively, with slits set at 5 nm.

### Crystallization and structure determination

The β2-m Trp95Leu mutant was crystallized under the same conditions used for the β2-m Trp60Val mutant [12]. X-ray diffraction data were collected using the crystallization mother liquor (19-20% PEG 4000, 20% glycerol, 0.2 M ammonium acetate, 0.1 M MES pH 6.0) as cryoprotectant on an ID14-1 crystallography beamline, at 100 K (ESRF, Grenoble, Switzerland). Diffraction data were processed using MOSFLM and SCALA [49,50]. β2-m Trp95Leu structure solution was achieved by molecular replacement, using MOLREP [51] and the wild-type β2-m atomic coordinates (PDB entry 1LDS) as search model. The structure was then refined with REFMAC5, at 1.57 Å resolution, applying the maximum likelihood residual, anisotropic B-factor refinement, riding hydrogen atoms, and atomic displacement parameter refinement using the 'tls' method [52]. Model building and structure analysis was performed with COOT [53]. Figure 8 was prepared using Pymol (http://www.pymol.org). Atomic coordinates and structure factors for β2-m Trp95Leu have been deposited with the Protein Data Bank, with accession code 3QDA.

## Authors' contributions

The study was conceived, designed and supervised by VB. MS, MS and PM contributed to the experimental design. SR and PM performed the equilibrium denaturation, folding kinetics and fibrillogenesis experiments. NB and RB performed the sequence alignment and phylogenetic analysis. GE, SR and MB performed the structural and crystallisation studies. IZ, ML, CS and MM produced and purified the recombinant proteins. VB wrote the paper and SR, PM, NB and RB contributed to the draft. All authors read and approved the final manuscript.

## Supplementary Material

Additional file 1 **Reconciliation between reduced species tree and reduced gene tree, conducted by GeneTree 1.3.0**. Good topology agreement is demonstrated between the reduced versions of the species and gene trees. Click here for file

Additional file 2 **Reconciled tree for the reduced β2-microglobulin gene family**. The reduced gene tree was reconciled using GeneTree with the species tree. Squares indicate duplication events, grey lines indicate absent genes, either lost from those species or not yet sequenced. Click here for file

Additional file 3 **Reconciled tree for the whole β2-microglobulin gene family**. The gene tree was reconciled with the species tree using Notung 2.6 (parameter values: 0.9 for losses, 1.35 for duplications, and no cost for conditional duplications). The D/L Score has been used to infer the root of a gene tree. Red squares indicate duplication events, grey lines indicate absent genes, either lost from those species or not yet sequenced. Edges with the minimum root score are highlighted in red and edges with near optimal scores are in pink. Click here for file
