# Supplementary figures and images for: The two tryptophans of β2-microglobulin have distinct roles in function and folding and might represent two independent responses to evolutionary pressure
Source: BMC Evol Biol. 2011 Jun 10;11:159. doi: 10.1186/1471-2148-11-159 (PMC3124429; doi:10.1186/1471-2148-11-159)

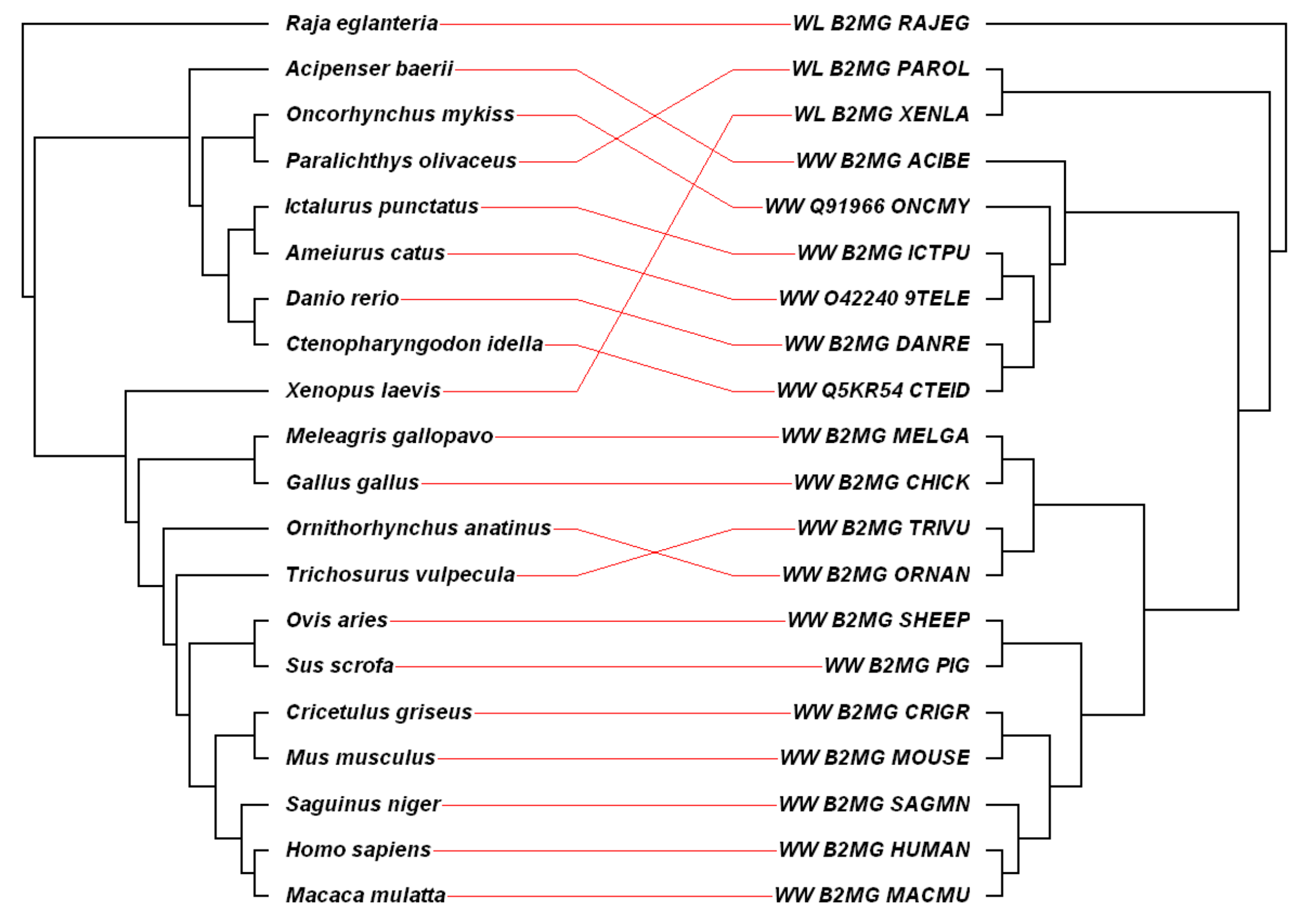

Supplement: Additional file 1 — Reconciliation between reduced species tree and reduced gene tree, conducted by GeneTree 1.3.0. Good topology agreement is demonstrated between the reduced versions of the species and gene trees. [file 1471-2148-11-159-S1.PNG]

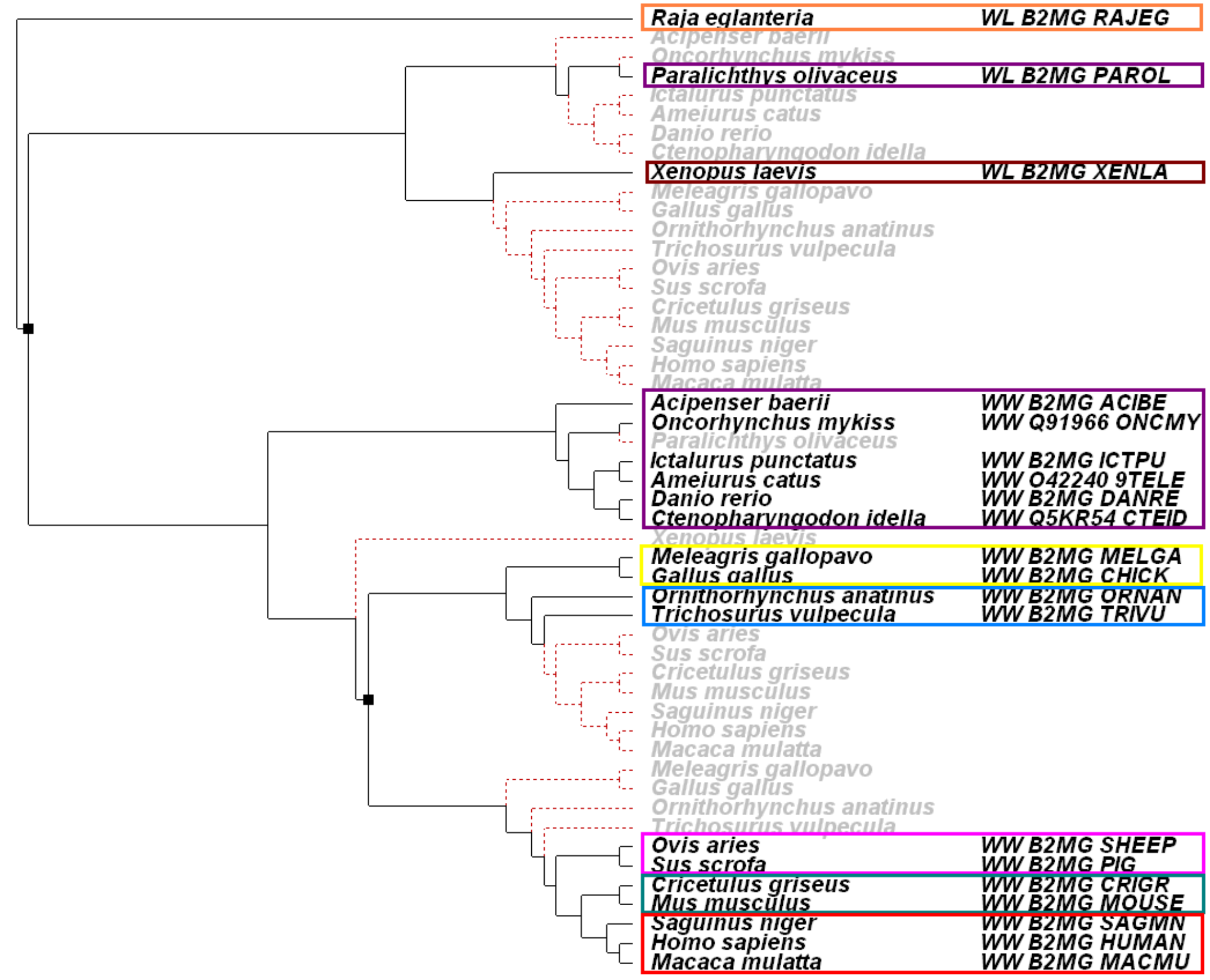

Supplement: Additional file 2 — Reconciled tree for the reduced β2-microglobulin gene family. The reduced gene tree was reconciled using GeneTree with the species tree. Squares indicate duplication events, grey lines indicate absent genes, either lost from those species or not yet sequenced. [file 1471-2148-11-159-S2.PNG]

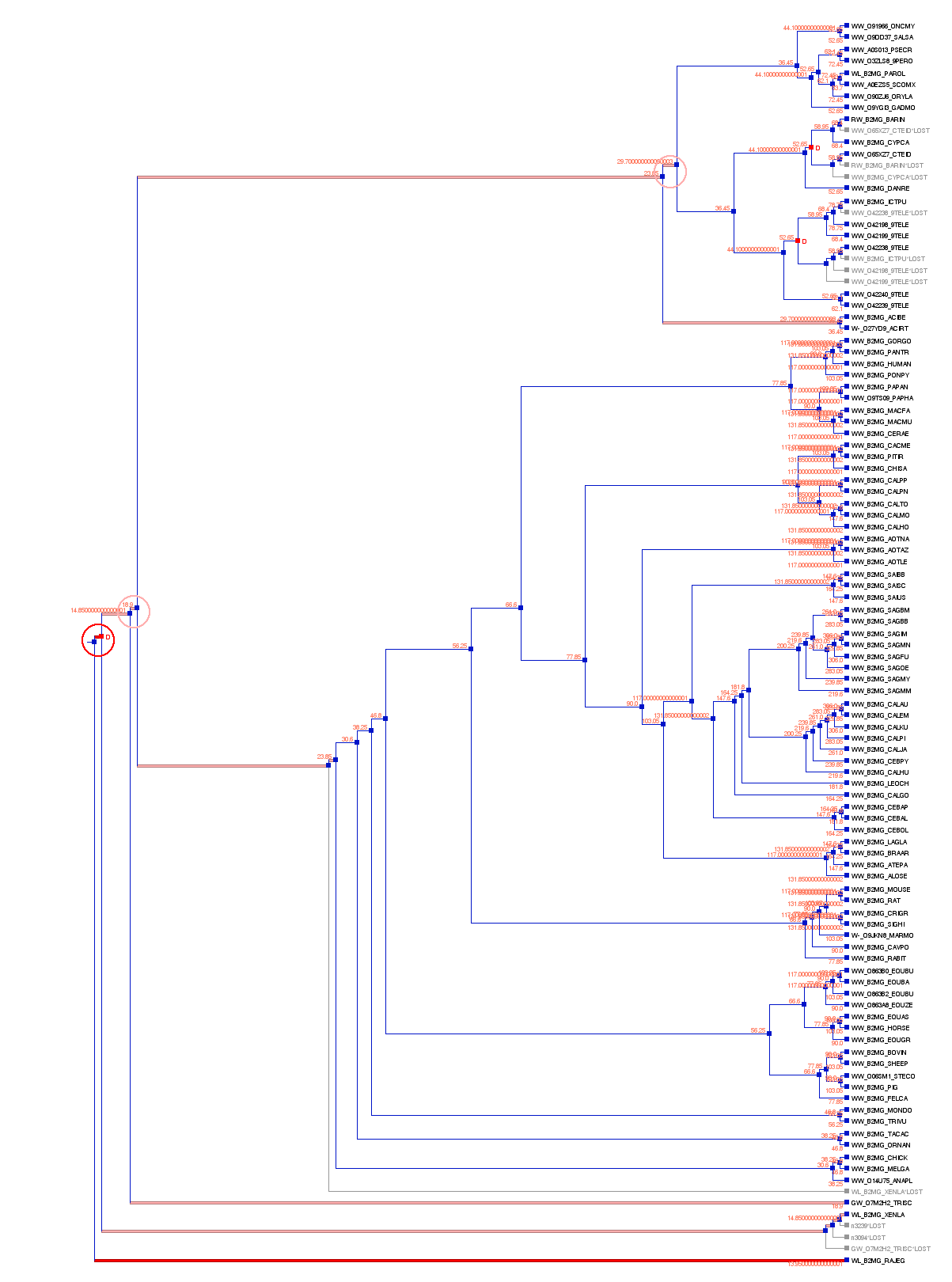

Supplement: Additional file 3 — Reconciled tree for the whole β2-microglobulin gene family. The gene tree was reconciled with the species tree using Notung 2.6 (parameter values: 0.9 for losses, 1.35 for duplications, and no cost for conditional duplications). The D/L Score has been used to infer the root of a gene tree. Red squares indicate duplication events, grey lines indicate absent genes, either lost from those species or not yet sequenced. Edges with the minimum root score are highlighted in red and edges with near optimal scores are in pink. [file 1471-2148-11-159-S3.PNG]
